# Evaluating Inhibition of the Epidermal Growth Factor (EGF)-Induced Response of Mutant MCF10A Cells with an Acoustic Sensor

**DOI:** 10.3390/bios2040448

**Published:** 2012-11-13

**Authors:** Marcela P. Garcia, Ammar Shahid, Jennifer Y. Chen, Jun Xi

**Affiliations:** 1 Department of Chemistry, Drexel University, 3141 Chestnut Street, Philadelphia, PA 19104, USA; E-Mails: mpg36@drexel.edu (M.P.G.); jyc29@drexel.edu (J.Y.C.); 2 Department of Biology, Drexel University, 3141 Chestnut Street, Philadelphia, PA 19104, USA; E-Mail: as954@drexel.edu

**Keywords:** QCM-D, biosensors, label free, EGFR, cell adhesion, cell signaling, cytoskeleton, inhibition, cancer development, drug screening

## Abstract

Many cancer treatments rely on inhibition of epidermal growth factor (EGF)-induced cellular responses. Evaluating drug effects on such responses becomes critical to the development of new cancer therapeutics. In this report, we have employed a label-free acoustic sensor, the quartz crystal microbalance with dissipation monitoring (QCM-D), to track the EGF-induced response of mutant MCF10A cells under various inhibitory conditions. We have identified a complex cell de-adhesion process, which can be distinctly altered by inhibitors of signaling pathways and cytoskeleton formation in a dose-dependent manner. The dose dependencies of the inhibitors provide IC_50_ values which are in strong agreement with the values reported in the literature, demonstrating the sensitivity and reliability of the QCM-D as a screening tool. Using immunofluorescence imaging, we have also verified the quantitative relationship between the Δ*D*-response (change in energy dissipation factor) and the level of focal adhesions quantified with the areal density of immunostained vinculin under those inhibitory conditions. Such a correlation suggests that the dynamic restructuring of focal adhesions can be assessed based on the time-dependent change in Δ*D*-response. Overall, this report has shown that the QCM-D has the potential to become an effective sensing platform for screening therapeutic agents that target signaling and cytoskeletal proteins.

## 1. Introduction

It is well known that the epidermal growth factor receptor (EGFR) regulates cell growth, proliferation, motility and differentiation through its downstream signaling pathways [1,2]. These downstream pathways such as the mitogen-activated protein kinase/extracellular signal-regulated kinase (MAPK/ERK) pathway [3], the phosphoinositide 3-kinase (PI3K) pathway [4], and the phospholipase C (PLC) pathway [5], are activated when epidermal growth factor (EGF) binds to the extracellular domain of EGFR. An abnormality in the EGFR system, such as overexpression and/or mutation of EGFR, increased production of the ligands (e.g., EGF) of EGFR, or downregulation of EGFR, can interfere with the tight regulation of these signaling pathways [6] and lead to the development of epithelial malignancies in humans such as cancers [7]. A better understanding of the effects of abnormalities in the EGFR system on cells will contribute to the understanding of the role of these abnormalities in cancer development, which, in turn, can lead ultimately to more effective cancer diagnosis, treatment, and prognosis [8].

Conventional approaches to the study of cellular responses to EGFR signaling rely on high spatial resolutions of radioactive or fluorescent labels to track the location, trafficking, and organization of one or more types of signaling molecules in a specific pathway. Such approaches have been successful in identification of some of the main activators, effectors, enzymes, and substrates in EGFR signaling [9]. However, because of the complexity of the signaling network and intracellular dynamics [10,11], information on specific components of a signaling pathway usually fails to provide the functional response of the whole cell. In addition, the temporal resolution of these label-based approaches is not high, thereby limiting the amount of information that can be obtained on the dynamics of cell signaling. Incorporation of various labels may also create a non-native and physiologically irrelevant cellular environment, which could potentially lead to ambiguous results [12,13,14]. 

These problems can be circumvented with the use of label-free biosensor technologies, which are capable of providing integrated and phenotypic readouts at the whole cell level [15]. Compared with label-based technologies, label-free biosensor technologies based on either optical or non-optical sensing platforms [16,17], are, for the most part, non-invasive, and capable of real-time measurement of cell signaling kinetics with high temporal resolution and high sensitivity [18]. The primary examples of optical, label-free sensing platforms are resonant waveguide grating (RWG) [19] and surface plasmon resonance (SPR) [20], both of which use a surface bound evanescent wave to determine the refractive indices of biomolecules [21]. Meanwhile, electrical impedance, acoustic resonance, microcantilevers, field effect nanowires and differential calorimetry belong in the category of non-optical, label-free biosensors [16]. The benchmark technology among these non-optical platforms is electrical impedance, which uses its sensitivity to ionic movement under electric fields to indicate morphological changes in a layer of cells [22]. 

Over the years, the applications of label-free biosensors in cell biology, including aspects of cell adhesion, cell barrier functions, cell signaling, and viral infection, have been steadily increasing [15]. In particular, the detection of functional responses of cells to EGFR signaling has attracted tremendous interests due to the biological significance of EGFR in cancer diagnosis, treatment, and prognosis. RWG, SPR, electrical impedance, and acoustic resonnance-based detection methods for this application have all been reported [23,24,25,26]. 

The quartz crystal microbalance with dissipation monitoring (QCM-D) is an acoustic resonance-based sensor that measures the changes in mass (from the change in vibrational frequency Δ*f*) and mechanical properties (from the change in energy dissipation factor Δ*D*) of a layer of biomolecules attached to the surface of an oscillating AT-cut quartz crystal [27]. The correlation between Δ*D* and Δ*f* can be used as an indicator of the cell-substrate interaction [28]. Like other label-free biosensors, the QCM-D is non-invasive and highly sensitive, and has a unique capability to simultaneously assess changes in mass and energy dissipation of the material that is coupled to the surface of the sensor crystal. This capability makes it a useful tool in the field of material and biological sciences [29,30]. 

In recent years, the QCM-D has become particularly attractive in the field of cell biology for its ability to monitor the interaction between cells and the surface to which they are attached [31,32,33,34,35,36] and determine the kinetics of cell attachment and spreading [27,37,38]. Applications of the QCM-D to the study of functional response of cells to receptor-mediated cell signaling have also begun to emerge as a result of our own research efforts [23,39,40]. We have successfully measured the short-term responses of human epidermoid carcinoma A431 cells to EGFR-mediated signaling based on real-time monitoring of changes in the dissipation factor (Δ*D*) and frequency responses (Δ*f*) [23]. We have also demonstrated the capability of the QCM-D in tracking the functional responses of both wildtype and mutant MCF10A cells during the EGF-induced cell de-adhesion (*i.e*., the reverse process of adhesion) [39,41]. A correlation between the time-dependent Δ*D*-response of the QCM-D and the level of focal adhesions of the cells has been established in those studies [39]. Based on this strong correlation, we have examined the regulation of this dynamic de-adhesion by the downstream pathways of EGFR signaling including the PI3K, MAPK/ERK, and PLC pathways [39].

The present paper reports an investigation of the EGF-induced de-adhesion of mutant MCF10A cells from the surface to which they are attached. Compared with wildtype MCF10A cells, these mutant cells possess a much higher level of EGFR [42], a situation that mimics the abnormal levels of EGFR in many types of solid tumors [43]. Such an abnormality has been linked to dysregulated cellular functions, including cell adhesion, which is part of pathological processes [44,45] that can promote tumor invasion and metastasis [6,46]. Finding an effective way to suppress tumor migration by limiting the EGF-induced cell de-adhesion might contribute to the development of a potentially more effective cancer treatment. Thus, the focus of this study was on evaluation of the inhibition of the EGF-induced de-adhesion in mutant MCF10A cells. The QCM-D was used to track the real-time responses of cells during this dynamic process. This study shows that the EGF-induced cell de-adhesion can be distinctly altered by inhibitors of signaling pathways (PD158780 for the EGFR activation, LY294002 for the PI3K pathway, U73122 for the PLC pathway, and L-779450 for the MAPK/ERK pathway) and cytoskeleton formation (cytochalasin D for actin polymerization) in a dose-dependent manner. The potency of each inhibitor as determined by means of the QCM-D corresponds well with the value reported in the literature. This strong agreement strongly suggests that the QCM-D has the potential to become an effective sensing platform for screening therapeutic agents that target signaling and cytoskeletal proteins.

## 2. Experimental Section

### 2.1. Reagents

Dulbecco’s modified Eagle’s medium and Ham’s F-12 nutrient mixture (DMEM/F12), horse serum, antibiotics, trypsin-EDTA, HEPES buffer, and HBSS buffer were purchased from Invitrogen. Human epidermal growth factor was purchased from Peprotech. Hydrocortisone, cholera toxin, and insulin were obtained from Sigma-Aldrich. PD158780 was purchased from EMD Bioscience. LY294002 and U73122 were purchased from Cayman Chemical Company, cytochalasin D was purchased from Enzo Life Sciences, and L-779450 (RafKinase Inhibitor IV) was purchased from Calbiochem.

### 2.2. Cell Culture

Mutant MCF-10A cells that overexpress EGFR were derived from stable transfection [42]. They were grown in T75 Corning flasks and maintained under a humidified atmosphere at 37 °C and 5% CO_2_ in DMEM/F12 medium containing 5% horse serum, 20 ng/mL EGF, 0.5 µg/mL hydrocortisone, 50 ng/mL cholera toxin, 10 µg/mL insulin, 100 IU/mL penicillin, and 100 µg/mL streptomycin. The cells were harvested at 95% confluency with the treatment of 0.25% trypsin-EGTA at 37 °C for 10 min. The cell medium containing trypsin was then removed and the cells were re-suspended in growth medium for plating on sensors and/or coverslips. The maximum number of cell passage used for this study was 12. The Stratagene mycoplasma plus PCR primer set was used to detect mycoplasma contamination.

### 2.3. QCM-D Measurements

A QCM-D (E4, Q-Sense) was used to record changes in energy dissipation factor (∆*D*) as a function of time at the odd overtone (n = 3) as previously described [39]. The sensors were prepared as follows: The QCM-D sensor crystals (gold-surfaced) were first washed with water and ethanol, then were dried under flowing nitrogen gas, and finally were exposed to UV-ozone for 20 min. The sensors were then stored in the tissue culture hood under UV-light for an additional 30 min. The virtually identical behavior of MCF10A cells on both gold-surfaced and glass-surfaced crystals [39] allowed us to proceed to compare the Δ*D* responses of the cells on gold-surfaced crystals with the fluorescence images of the cells on glass-surfaced coverslips. The sensors were then placed in a 12-well tissue culture plate along with mutant MCF-10A cells that had been harvested from the T75 culture flask. The cells were allowed to grow on the sensors under a humidified atmosphere at 37 °C and 5% CO_2_.

When they reached 95% confluency, the cells were starved in serum free medium for 18 h. Each sensor crystal with a cell layer was then mounted in an open module (Q-sense) and incubated in 400 µL of the assay buffer (20 mM HEPES in HBSS buffer, pH 7.2) at 37 °C. After the stable baselines were established for all four sensors, the cells were then incubated in 400 µL of the inhibitor solution at 37 °C for 40 min. Then the inhibitor solution was replaced with the same volume of 10 nM EGF in the assay buffer containing the same concentration of the inhibitor and the cells were incubated for 3 h. 

### 2.4. Fluorescence Imaging

Cells were seeded on coverslips and allowed to grow to 95% confluency in a humidified atmosphere at 37 °C and 5% CO_2_. The cells were then starved in serum-free medium for 18 h. Prior to immunostaining, the cells on coverslips were incubated in 1 mL of the assay buffer at 37 °C for 1 h, then in the inhibitor solution for 40 min, and finally in 1 mL of 10-nM EGF solution containing the same concentration of the inhibitor for 3 h. For immunostaining, the cells were fixed in a solution of 0.1% Triton X-100 and 3% paraformaldehyde in PHEM buffer (60 mM PIPES, 25 mM HEPES, 10 mM EGTA, 2 mM MgCl_2_ and pH 6.9) at room temperature for 20 min. The fixed cells were first treated with monoclonal mouse anti-vinculin antibody (Invitrogen) at a concentration of 1:200 in blocking buffer (PBS with 2% BSA) at room temperature for 120 min, and then with Alexafluor 546 goat anti-mouse (Invitrogen) antibody at a concentration of 1:200 in staining buffer (PBS with 2% BSA) at room temperature for 60 min. The coverslips with stained cells were mounted in Vectashield medium DAPI (Vector Laboratories, Inc.) and were imaged with an inverted fluorescence microscope (Zeiss Axioplan 2). All images were processed with the use of Slidebook 5.0 software (Intelligent Imaging Innovations). 

### 2.5. Data Analysis

The dose-response curve was created by plotting the average amplitudes (±1 std. dev.) of the ∆*D*-responses *versus* inhibitor concentrations. Each amplitude value, defined as the absolute value of the difference between the experimental value and the control value, was taken at 40 min of the ∆*D*-response. IC_50_ value was calculated with the use of PSI-Plot (Poly Software International) by fitting the data to the following equation:

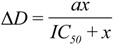
(1)
where *x* is the concentration of the inhibitor. *a* corresponds to the maximum ∆*D*-response, which can be determined through curve fitting. The use of the log functional plot or sigmoid plot for this analysis would not significantly alter the resulting IC_50_ values.

### 2.6. Fluorescence Quantitation

The areal density of focal adhesions was quantified with ImageJ software (http://rsb.infor.nih.gov/ij/). For each image, the background intensity was first determined from the fluorescence of a selected empty area. Then the density of focal adhesions of a selected cell was obtained by subtracting the background intensity from the total fluorescence intensity (IntDen) of immunostained vinculin. Ten randomly selected cells were analyzed for each image and the standard error of the mean (SEM) was computed. The student t-test was used for statistical analysis of the data set.

## 3. Results and Discussion

### 3.1. Overview

In our previous study, we had been able to employ the QCM-D to track the EGF-induced cellular responses of confluent monolayers of human epithelial breast MCF-10A cells. The time-dependent Δ*D*-responses measured by the QCM-D were found to be correlated with dynamic changes in areal density of focal adhesions of the cells [39]. Since the areal density of focal adhesions is quantitatively related to the strength of cell adhesion [47,48,49,50], we then used the Δ*D*-response as an indicator of the strength of cell-substrate adhesion to assess the EGF-induced cell de-adhesion in wildtype MCF10A cells [39]. The cells exhibit a complex de-adhesion process, consisting of de-adhesion, transition, and re-adhesion [39]. In these studies, as in the present study, we used various inhibitors to modulate the EGF-induced Δ*D*-responses in order to gain mechanistic insights into the regulation of cellular responses.

In the present study, we sought to assess the potential of using the QCM-D as a sensing platform for drug screening. Due to the limited throughput of the QCM-D, where only four independent experiments can be run simultaneously, the QCM-D is far from ready for drug screening. Therefore, the focus of the present study was on sensitivity and reliability. We first evaluated the dose response for inhibition of the EGF-induced cell de-adhesion process in mutant MCF10A cells that overexpress EGFR. We then verified the time dependence of the inhibition with immunofluorescence imaging. Finally, we determined the potency of each inhibitor and compared its experimental value with the literature value. All QCM-D experiments were conducted at 37 °C, and stable baselines were achieved prior to the addition of inhibitors. For each inhibitor, we carefully selected six to seven different concentrations, which cover the range that allows the attainment of a reasonable dose-response curve and a reliable IC_50_ value. Although variations often occur for cell-based assays, we made an effort to minimize the impact of any potential variations on the outcomes of the study by conducting QCM-D experiments with cells from at least three separate cultures and fluorescence imaging experiments with cells from two separate cultures. In addition, carefully designed control experiments were performed under individual conditions. For the dose response studies, the variations did not significantly alter the determination of IC_50_ values.

### 3.2. Inhibition of the EGF-Induced Time-Dependent Cellular Responses: QCM-D Measurements

**Figure 1:**
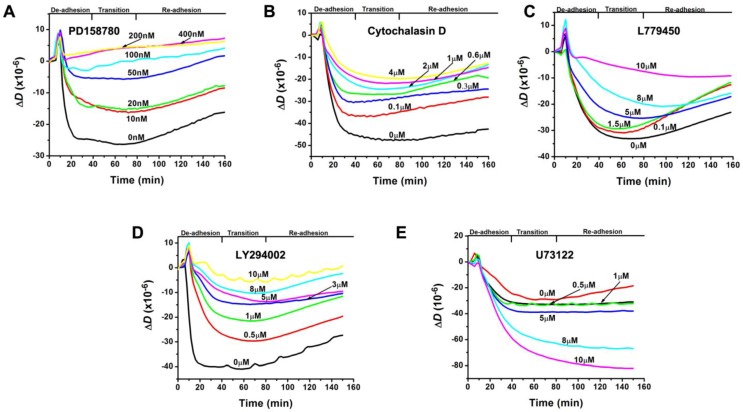
Real-time quartz crystal microbalance with dissipation monitoring (QCM-D) measurements (at the order of overtone *n* = 3) of the Δ*D*-responses of mutant MCF-10A cells to 10 nM epidermal growth factor (EGF) at 37 °C. The corresponding sequential EGF-induced de-adhesion processes were indicated. (**A**) The Δ*D*-responsesof the cells were suppressed by PD158780, a known inhibitor of EGFR tyrosine kinase, at various doses (0, 10, 20, 50, 100, 200, and 400 nM). (**B**) The Δ*D*-responsesof the cells were suppressed by cytochalasin D, a known inhibitor of actin polymerization, at various doses (0, 0.1, 0.3, 0.6, 1, 2, and 4 µM). (**C**) The Δ*D*-responsesof the cells were suppressed by L779450, a known inhibitor of Raf kinase in the mitogen-activated protein kinase/extracellular signal-regulated kinase (MAPK/ERK) pathway, at various doses (0, 0.1, 0.5, 5, 8, and 10 µM). (**D**) The Δ*D*-responsesof the cells were suppressed by LY294002, a known inhibitor of PI3K in the PI3K pathway, at various doses (0, 0.5, 1, 3, 5, 8, and 10 µM). (**E**) The Δ*D*-responsesof the cells were increased by U73122, a known inhibitor of phospholipase C (PLC)γ in the PLC pathway, at various doses (0, 0.5, 1, 5, 8, and 10 µM).

Figure 1(A) shows the results of a typical dose-response inhibition of the EGF-induced cellular response measured with the QCM-D. In this experiment, seven different doses (0, 10, 20, 50, 100, 200, and 400 nM) of PD158780, a potent inhibitor of EGFR tyrosine kinase [51] were introduced into confluent monolayers of mutant MCF-10A cells on QCM-D sensor crystals. After incubation at 37 °C for 40 min, each assay solution was replaced with a solution of 10 nM EGF. The Δ*D*-responses at the order of overtone, *n* = 3, were recorded at 37 °C for 3 h. Although the QCM-D is able to provide simultaneous measurements of both Δ*D*- and Δ*f*-responses, we focused on the Δ*D*-responses because it is a far more sensitive measure of the EGF-induced cellular response than the Δ*f*-response [39]. 

In Figure 1(A), the Δ*D*-response curve labeled 0 nM PD158780 represents the time-dependent profile of mutant MCF10A cells responding to 10 nM EGF without the presence of the inhibitor. Immediately upon the addition of EGF, this Δ*D*-response curve exhibits a sharp upward spike, which was an artifact of the mechanical perturbation caused by adding a liquid to the cell layer. After the initial spike, the Δ*D*-response curve shows a rapid decline that continues until ~25 min, which corresponds to a rapid cell de-adhesion. At ~40 min, the rate of this decline begins to decrease until the valley of the Δ*D*-response is reached at ~65 min. The response curve then maintains a virtually constant response from the valley for the next 15 to 20 min. The period from 40 to 80 min is considered to be the transition phase of the Δ*D*-response. After the transition, the Δ*D*-response begins a slow but steady increase for the next 80 min, which corresponds to cell re-adhesion. The response profile of mutant MCF10A cells typically shows a more rapid de-adhesion and slower re-adhesion compared with that of the wildtype cells [41].

In the presence of PD158780, the amplitude of each EGF-induced Δ*D*-response curve was substantially reduced, and the higher the concentrations of the inhibitor, the greater the reduction of the amplitudes of the EGF-induced Δ*D*-response. At the two highest concentrations (200 and 400 nM), the EGF-induced Δ*D*-responses were completely abolished, as evidenced by their nearly zero amplitudes (Figure 1(A)). All of these observed changes to the EGF-induced Δ*D*-responses indicate an essential role of the EGFR tyrosine kinase in regulation of cell de-adhesion.

Figure 1(B) shows the inhibitory effect of cytochalasin D on the EGF-induced Δ*D*-response. Cytochalasin D (CD), is a potent, cell-permeable inhibitor of actin polymerization and is capable of attenuating the remodeling of the actin filament [52]. When the cells were pretreated with CD, the EGF-induced Δ*D*-response was substantially suppressed (Figure 1(B)), confirming that cytoskeleton remodeling is associated with the observed Δ*D*-response to EGF. This further underscores the connection between cytoskeleton remodeling and cell signaling and trafficking in general [53,54].

Figure 1(C–E) show the results of the inhibition of downstream signaling pathways of EGFR including the MAPK/ERK, PI3K, and PLC pathways. These pathways, which are responsible for regulating the assembly and disassembly of focal adhesions that lead to changes in cell adhesion [55,56,57], have been the targets for therapeutic development in recent years [58,59,60]. To probe these pathways, we pretreated cells with L779450 [61], a potent cell-permeable inhibitor of Raf kinase in the MAPK/ERK pathway, with LY294002, a potent inhibitor of PI3K in the PI3K pathway [62], or with U73122, a potent inhibitor of PLCγ (an isotype of phospholipase) in the PLC pathway [63]. It is evident that the EGF-induced Δ*D*-responses were altered by the inhibitors in a dose-dependent manner (Figure 1(C–E)). For both L779450 (Figure 1(C)) and LY294002 (Figure 1(D)), the amplitudes of the EGF-induced Δ*D*-responses were reduced, suggesting that the MAPK/ERK and PI3K pathways are responsible for promoting the EGF-induced cellular response in mutant MCF-10A cells. These results are in line with the previous reports that the MAPK/ERK pathway is responsible for the EGF-induced disassembly of focal adhesion in fibroblasts [5] and the PI3K pathway may alter cell adhesion through its downstream effectors including small GTPase Rho A, and/or through crosstalk with the MAPK/ERK pathway [55]. For U73122, the amplitudes of the EGF-induced Δ*D*-responses were not reduced. Instead, they increased as the concentration of U73122 increased (Figure 1(E)), suggesting an enhanced cell de-adhesion upon the inhibition of the PLC pathway, a pathway known to play an important role in EGF-mediated cell adhesion and motility [64].

Interestingly, the inhibition of each pathway led to distinct features of the time-dependent dose-response profiles, shown in Figure 1(C–E). For example, when the MAPK/ERK pathway was inhibited by L779450, the portions of the EGF-induced response curves corresponding to the initial rapid de-adhesion (first 20 min) remained close to each other for most inhibitor concentrations (Figure 1(C)). By contrast, all inhibitory response curves were well separated from each other during the same period of time (first 20 min) when the PI3K pathway was inhibited by LY294002 (Figure 1(D)). While the re-adhesion still occurred when either the MAPK/ERK or PI3K pathway was inhibited (Figure 1(C,D)), re-adhesion was clearly absent when the PLC pathway was inhibited (Figure 1(E)). These distinct features of the inhibition profiles can be regarded as the “signatures” of the cellular response of individual pathways, *i.e.*, a unique form of the pathway-dependent phenotypic response [65,66,67]. These signatures could potentially be very informative by providing mechanistic insights into the regulation of the EGF-induced cell de-adhesion in mutant MCF10A cells.

### 3.3. Inhibition of the EGF-Induced Time-Dependent Cellular Responses: Fluorescence Imaging

Label-free biosensors are capable of producing real-time, measurable signals by capturing time-dependent cellular responses, which often result from more than one of many possible changes that relate to cellular morphology, adhesion, ion distribution, and mass distribution [16,17,67]. Because these signals are the results of the collected effects within cells, individual contributing molecular components and their associated cellular processes are often difficult to identify. Thus, the label-free biosensor signals are commonly referred to as “black box” assay readouts [68].

In the previous work, we had examined the time-dependent cell de-adhesion in response to EGF with fluorescence imaging. We were able to establish a quantitative relationship between the Δ*D*-response obtained from the QCM-D and the level of focal adhesions, quantified with measurements of the areal density of immunostained vinculin [39]. To our knowledge, this is one of the few studies done to make a quantitative connection between a cell-based biosensor signal and a specific cellular component and its associated cellular process. Following the same strategy in the present study, we intended to verify the inhibitory effect of each inhibitor on mutant MCF10A cells and further establish the connection between the time-dependent Δ*D*-response and the dynamic restructuring of focal adhesions.

**Figure 2:**
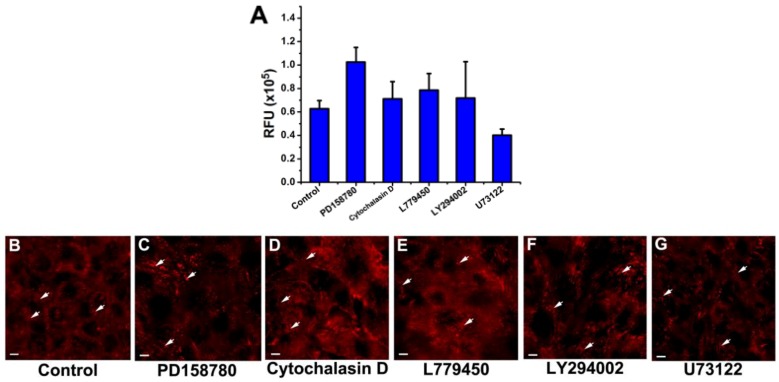
Fluorescence images of immunostained vinculin within focal adhesions of mutant MCF10A cells in response to 10 nM EGF. Examples of focal adhesions are indicated with arrows. Scale bar: 10 µm. The cells had been pretreated with an inhibitor described in (B–G). (**A**) Quantitation of the areal densities of stained vinculin in relative fluorescence units (RFU) as a measure for the level of focal adhesions (mean ± SEM; n = 10) in (B–G). (**B**) The control without the presence of an inhibitor. (**C**) 100 nMPD158780. (**D**) 1 µM cytochalasin D. (**E**) 10 µM L77945. (**F**) 10 µM LY294002. (**G**) 5 µM U73122. The difference in fluorescence intensities between the control and each inhibited sample is significant as indicated by P < 0.001 for all five inhibited samples.

First we examined the inhibitory effect of each inhibitor on the EGF-induced changes in level of focal adhesions. Figure 2(B–G) show fluorescence images of immunostained vinculin within focal adhesions of mutant MCF10A cells. Each image was taken from the cell sample that had been pretreated with a specific inhibitor (or the assay buffer for the control) for 40 min and then exposed to 10 nM EGF for 60 min. The overall comparison based on the quantitation of fluorescence intensities of stained vinculin is shown in Figure 2(A). With the presence of the inhibitors including PD158780 (100 nM, Figure 2(C)), cytochalasin D (1 µM, Figure 2(D)), L779450 (10 µM, Figure 2(E)), and LY294002 (10 µM, Figure 2(F)), the cells exhibit slightly higher fluorescence intensities of vinculin compared to the control which does not have any inhibitors (Figure 2(B)). These results indicate that these inhibitors enhanced the cell adhesion by suppressing the EGF-induced de-adhesion, which is consistent with the observed reduction of the amplitudes of the Δ*D*-responses in Figure 1(A–D). For the sample that had been pretreated with 5 µM U73122 (Figure 2(G)), the cells exhibit a slightly lower fluorescence intensity compared to the control (Figure 2(A)), which is consistent with the increasing amplitudes of the Δ*D*-responses in the presence of U73122 (Figure 1(E)), a sign of an enhanced cell de-adhesion. 

**Figure 3:**
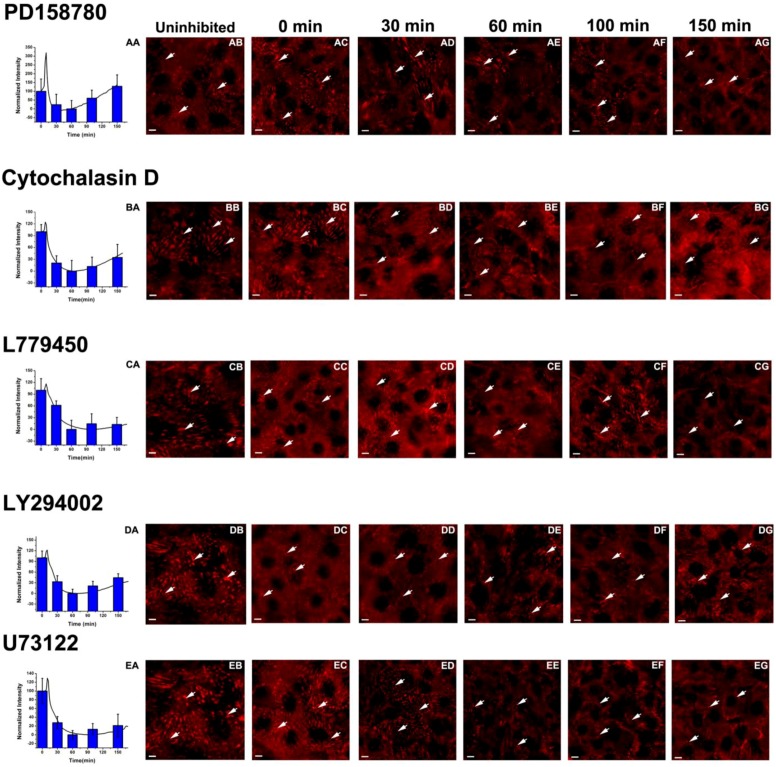
Time dependent relationship between changes in energy dissipation factor and vinculin immunofluorescence staining within focal adhesions of mutant MCF10A cells in response to 10 nM EGF. Examples of focal adhesions are indicated with arrows. Scale bar: 10 µm. The cells had been pretreated with the following inhibitors: 100 nM PD158780 (row **A**), 1 µM cytochalasin D (row **B**), 10 µM L779450 (row **C**), 10 µM LY294002 (row **D**), and 5 µM U73122 (row **E**). (**AA**), (**BA**), (**CA**), (**DA**), and (**EA**) Quantitation of the areal densities of stained vinculin in relative fluorescence units (RFU) as a measure of focal adhesions (mean ± SEM; n = 10). A strong correlation is shown between the normalized Δ*D*-responseand the normalized RFU of focal adhesions. To ensure that the values of RFU and Δ*D* could be compared, each of the values was normalized, *i.e.*, was divided by the range covered. For each quantity, the range was taken as the highest value (at 0 min) minus the lowest value (at 60 min). All correlations are highly statistically significant (p < 0.005). In each of rows (A) to (E), column (B) shows the fluorescence images of focal adhesions in a monolayer of cells prior to inhibition, labeled as uninhibited. Columns (C) to (G) show the fluorescence images of focal adhesions in a monolayer of cells after being exposed first to the inhibitor for 40 min, then to 10 nM EGF for: (**C**) 0 min, (**D**) 30 min, (**E**) 60 min, and (**F**) 100 min, and (**G**) 150 min.

Next we investigated the time-dependent effect of each inhibitor by focusing on changes in areal density of focal adhesions (Figure 3). Figure 3(AC–AG) show fluorescence images of immunostained vinculin within focal adhesions of mutant MCF10A cells that had been pretreated with 100 nM PD158780 and then exposed to 10 nM EGF for various times (0, 30, 60, 100, and 150 min). Because of the high fluorescence background, some of the images in Figure 3 look brighter than others, even though the intensities of immunostained vinculin in those images may not be necessarily higher than others. The appearance of dark holes enclosed within bright boundaries was probably due to immunostained vinculin at sites of cell-cell contact. Prior to exposure to EGF (0 min), numerous prominent focal adhesions are present as short bright streaks of vinculin in both the central regions and the peripheries of the cells that had been pretreated with 100 nM PD158780 (Figure 3(AC)). Upon exposure to 10 nM EGF for 30 min, the cells exhibit fewer, smaller, and less intense spots of stained vinculin (Figure 3(AD)), indicating a lowered level of focal adhesions. A 60-min exposure to EGF further diminished the spots of stained vinculin in size and number, as shown in Figure 3(AE). However, a longer exposure to EGF, e.g., 100 min, did not cause any further diminution of stained vinculin compared with the 60 min exposure. In fact, they show a slight increase in both size and number, which is indicative of an increase in level of focal adhesions (Figure 3(AF)). After a 150 min exposure to EGF, stained vinculin spots (Figure 3(AG)) become even more noticeable compared with the 100-min exposure. Overall, when exposed to EGF, a monolayer of mutant MCF10A cells underwent time-dependent restructuring of focal adhesions, which corresponds to de-adhesion, transition (around 60 min), and re-adhesion. This pattern is consistent with the one revealed with the QCM-D measurements in Figure 1(A). The cells in the presence of other inhibitors in this study also showed similar patterns to that of PD158780 (Figure 3). 

For each inhibitor, the levels of focal adhesions are quantified and summarized as a function of time in bar graph form in Figure 3(AA,BA,CA,DA,EA), respectively. The time-dependent changes in level of focal adhesions were fit with the corresponding Δ*D*-response curve. Reasonably good fits are obtained for all five inhibitors, confirming the correlation between the magnitude of the Δ*D*-response and the level of focal adhesions, and suggesting that the change in cell adhesion, more specifically, the restructuring of focal adhesions can be assessed based on the time-dependent change in Δ*D*-response. 

### 3.4. Inhibition of the EGF-Induced Time-Dependent Cellular Responses: Potencies of the Inhibitors

By far we have shown that the QCM-D has the capability of measuring the inhibitory effects of small molecule inhibitors on the EGF-induced cellular response. To have a more quantitative assessment of the effectiveness of the QCM-D, we determined the IC_50_ value for each of the five inhibitors. By comparing the experimental value with the reported value, we could assess the sensitivity and particularly the reliability of the QCM-D as a screening tool. 

**Figure 4:**
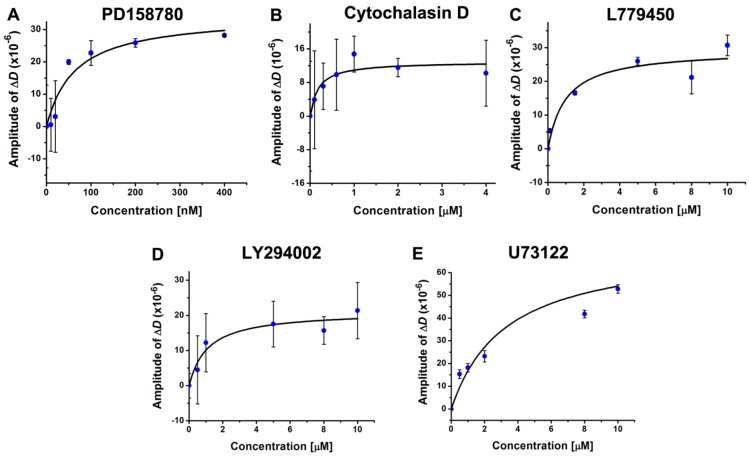
The amplitudes of EGF-induced Δ*D*-responsesat 40 min as a function of inhibitor concentrations.The amplitude is defined as the absolute value of the difference between the experimental value and the control value. The data, derived from the average of at least three sets of independent data, was fit with the dose-response function. The resulting IC_50_ values are also listed in Table 1. (**A**) PD158780. IC_50_ = 64 ± 30 nM. (**B**) cytochalasin D. IC_50_ = 0.18 ± 0.11 µM. (**C**) L779450. IC_50_ = 1.0 ± 0.6 µM. (**D**) LY294002. IC_50_ = 1.1 ± 0.5 µM. (**E**) U73122. IC_50_ = 2.5 ± 0.9 µM.

The IC_50_ value for each inhibitor was derived based on its dose dependence determined by fitting the amplitudes of the Δ*D*-response values at 40 min as a function of the inhibitor concentrations (Figure 4). These values obtained from the QCM-D measurements along with the values reported in the literature are listed in Table 1. The reported IC_50 _value for cytochalasin D was determined based on its inhibitory effect on the mechanical properties of the cell sample [69]. All other values were determined based on the *in vivo* inhibitory effects on the target enzymes in cell samples [62,70,71,72]. It is apparent that the experimental values are in strong agreement with the reported values (Table 1), which strongly supports the notion that the QCM-D has the sensitivity and reliability to be potentially utilized as a sensing platform for drug screening. To achieve this goal, increasing the throughput capacity of the QCM-D would be the next critical step. In addition, establishing the technical advantage of the QCM-D compared to other sensor technologies would also be important. 

**Table 1:** Comparison of IC_50_ values of the inhibitors.

Inhibitor	IC_50_ (QCM-D)	IC_50_ (literature)
PD158780	64 ± 30 nM	52 nM [70]
cytochalasin D	0.18 ± 0.11 µM	0.25 µM [69]
L779450	1.0 ± 0.6 µM	1 µM [71]
LY294002	1.1 ± 0.5 µM	1.4 µM [62]
U73122	2.5 ± 0.9 µM	1–2.1 µM [72]

## 4. Conclusions

In this report, we have employed a label-free acoustic sensor, the QCM-D, to track the EGF-induced response of mutant MCF10A cells under various inhibitory conditions. We have identified a complex cell de-adhesion process, which can be distinctly altered by inhibitors of signaling pathways and cytoskeleton formation in a dose-dependent manner. The dose dependencies of the inhibitors provide IC_50_ values which are in strong agreement with the values reported in the literature, demonstrating the sensitivity and reliability of the QCM-D as a screening tool. Using immunofluorescence imaging, we have also verified the quantitative relationship between the Δ*D*-response and the level of focal adhesions under those inhibitory conditions. Such a correlation suggests that the dynamic restructuring of focal adhesions can be assessed based on the time-dependent change in Δ*D*-response. Overall, this report has shown that the QCM-D has the potential to become an effective sensing platform for screening therapeutic agents that target signaling and cytoskeletal proteins. 
